# Genetic polymorphisms and expression of Rhesus blood group RHCE are associated with 2,3-bisphosphoglycerate in humans at high altitude

**DOI:** 10.1073/pnas.2315930120

**Published:** 2023-12-26

**Authors:** Angelo D’Alessandro, Eric J. Earley, Travis Nemkov, Daniel Stephenson, Monika Dzieciatkowska, Kirk C. Hansen, Giampaolo Minetti, Benoit Champigneulle, Emeric Stauffer, Aurélien Pichon, Michael Furian, Samuel Verges, Steven Kleinman, Philip J. Norris, Michael P. Busch, Grier P. Page, Lars Kaestner

**Affiliations:** ^a^Department of Biochemistry and Molecular Genetics, University of Colorado Anschutz Medical Campus, Aurora, CO 80045; ^b^Research Triangle Institute International, Atlanta, GA 30329-4434; ^c^Department of Biology and Biotechnology, University of Pavia, Pavia 27100, Italy; ^d^Hypoxia Physiopathology laboratory (HP2), INSERM U1042, Grenoble Alpes University, Grenoble 38400, France; ^e^Laboratoire Interuniversitaire de Biologie de la Motricité (LIBM) EA7424, Université Claude Bernard Lyon 1, Lyon 69100, France; ^f^Université de Poitiers, Laboratoire MOVE, Poitiers 20296, France; ^g^Pulmonology Department, University of Zurich, Zürich 1008091, Switzerland; ^h^Department of Pathology and Laborarory Medicine, University of British Columbia, Victoria, BC V6T 1Z4, Canada; ^i^Vitalant Research Institute, San Francisco, CA 94105; ^j^Dynamics of Fluids, Experimental Physics, Saarland University, Saarbrücken 66123, Germany

**Keywords:** red blood cell, metabolomics, hypoxia

## Abstract

Red blood cell (RBC) metabolic reprogramming upon exposure to high altitude contributes to physiological human adaptations to hypoxia, a multifaceted process critical to health and disease. To delve into the molecular underpinnings of this phenomenon, first, we performed a multi-omics analysis of RBCs from six lowlanders after exposure to high-altitude hypoxia, with longitudinal sampling at baseline, upon ascent to 5,100 m and descent to sea level. Results highlighted an association between erythrocyte levels of 2,3-bisphosphoglycerate (BPG), an allosteric regulator of hemoglobin that favors oxygen off-loading in the face of hypoxia, and expression levels of the Rhesus blood group RHCE protein. We then expanded on these findings by measuring BPG in RBCs from 13,091 blood donors from the Recipient Epidemiology and Donor Evaluation Study. These data informed a genome-wide association study using BPG levels as a quantitative trait, which identified genetic polymorphisms in the region coding for the Rhesus blood group RHCE as critical determinants of BPG levels in erythrocytes from healthy human volunteers. Mechanistically, we suggest that the Rh group complex, which participates in the exchange of ammonium with the extracellular compartment, may contribute to intracellular alkalinization, thus favoring BPG mutase activity.

At any given time during the life of an adult individual, approximately 25 trillion red blood cells (RBCs) circulate in the bloodstream (1). Each RBC carries ~250 to 270 million copies of hemoglobin/cell to transport and deliver oxygen to all tissues, a cell-specific function that is essential to life (1). One of the generational breakthroughs in biochemistry has been the characterization of the mechanisms that modulate this very function by controlling hemoglobin dynamics (2): Allosteric regulation of hemoglobin conformational changes facilitates oxygen off-loading in response to hypoxia, a process that is favored by the stabilization of the tense, deoxygenated state of hemoglobin by the organic phosphate compound 2,3-bisphosphoglycerate (BPG) (3). Generation of BPG via the Rapoport–Luebering shunt comes from rerouting of glycolysis upon the activation of bisphosphoglycerate mutase (BPGM), the rate-limiting enzyme of this pathway. However, it has been noted that BPGM activity favors the synthase reaction at alkaline pH, while the phosphatase reaction is dominant under acidic conditions (4). The Bohr effect also participates in these processes, whereby low pH and high CO_2_ levels promote protonation of functional histidine residues on hemoglobin, thereby facilitating oxygen off-loading. Therefore, respiratory alkalosis from hyperventilation early following exposure to hypoxia could impair O_2_ off-loading; however, the effects of alkaline pH on 2,3 BPG synthesis offset this phenomenon.

Physiological management of hypoxic conditions is not completely understood. After decades of published literature on RBC metabolic adaptations in vitro, only recently have these reports been corroborated by in vivo evidence of increased BPG synthesis in response to high-altitude hypoxia (5). This observation fueled the testing of hypoxic storage conditions to boost BPG synthesis in stored RBCs for transfusion purposes, the rationale being that rapid BPG consumption under refrigerated storage conditions may ablate oxygen kinetics in the stored RBCs, thus decreasing transfusion efficacy in patients requiring transfusions (6).

Recently appreciated mechanisms of RBC metabolic regulation in hypoxia involve signaling via adenosine, sphingosine 1-phosphate, and transglutaminase-dependent pathways, and the competitive binding of deoxyhemoglobin and glycolytic enzymes to the N-terminus cytosolic domain of band 3 (7). However, the genetics underpinnings of the heterogeneity in RBC BPG levels remain incompletely elucidated.

## Methods

**Fig. 1.**
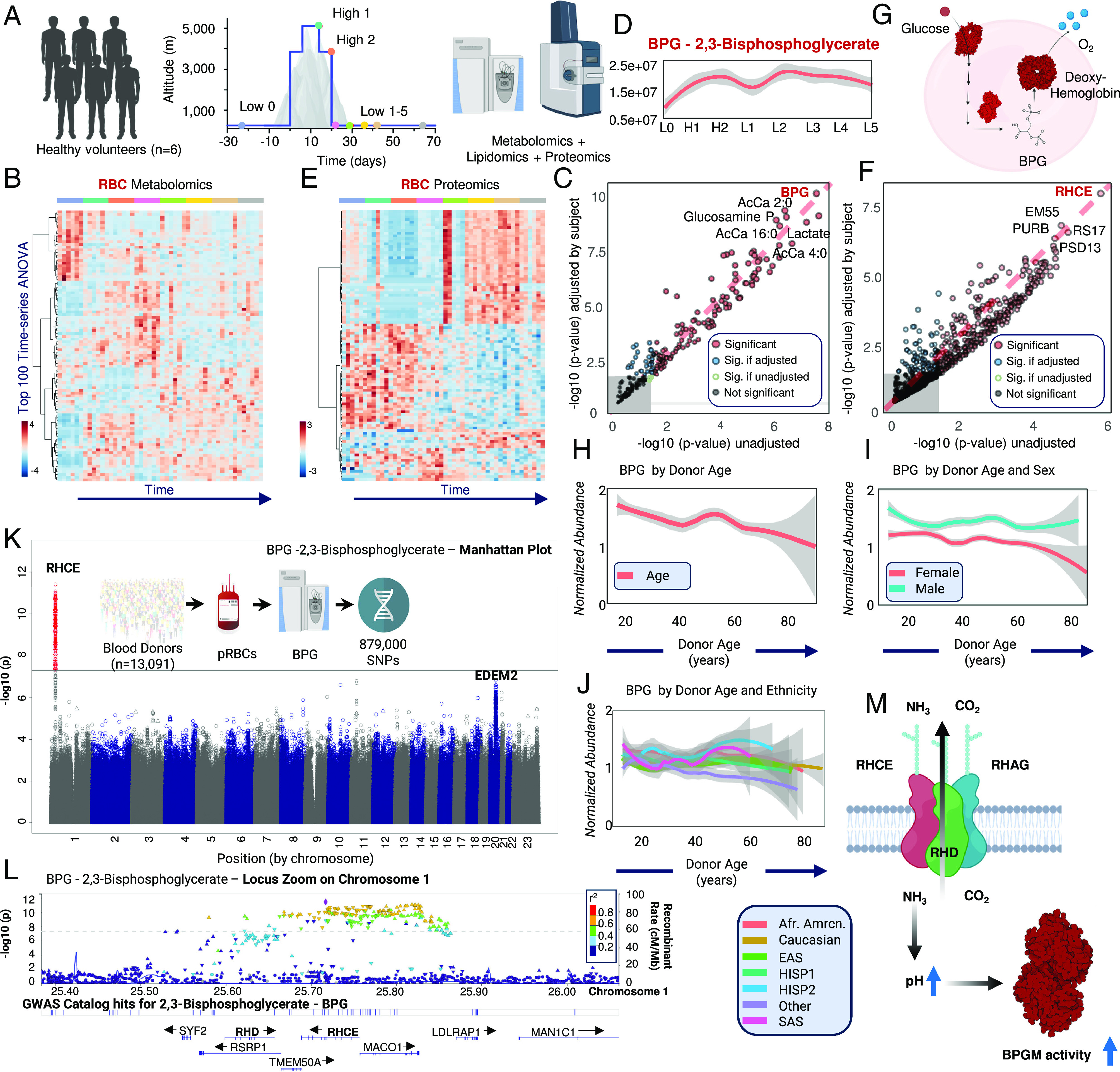
Multi-omics characterization of RBCs from the Expedition 5300 study (*A*). Time series-ANOVA and linear discriminant analyses identified top metabolites (*B* and *C*; line plot for BPG in *D*), and proteins (*E* and *F*) affected by ascent, acclimatization, and descent. In (*G*), BPG promotes oxygen off-loading by stabilizing the deoxygenated state of hemoglobin during acclimatization to high altitude. In packed RBCs from 13,091 volunteers from the REDS RBC Omics study, BPG levels are affected by donor age (*H*), sex (*I*), and ethnicity (*J*). mQTL analyses identified an association between BPG levels and SNPs on chromosome 1 (*K*), in the region coding for RHCE (locus zoom in *L*). In *L*, colors represent estimated linkage disequilibrium with the lead SNP (diamond–rs636889) based on the full (“ALL”) 1K genomes reference. Other shapes indicate: Framestop and splice (triangle), NonSynonymous (inverted triangle), and None-of-the-above (filled circle). Proposed mechanism (*M*).

To bridge this gap, as part of the “Expedition 5300” project, we performed metabolomics, lipidomics, and proteomics analyses of RBCs from six volunteers from lowland (approximately 250 m; time 0) to high altitude in La Rinconada, the highest city worldwide (Peru, 5,100 m—Fig. 1*A*) (8) This study was approved by the ethics committee of the Universidad Peruana Cayetano Heredia (Lima, Peru, IRB number: 00003251) and was conducted in accordance with the Declaration of Helsinki and upon signing of informed consent by all participants. Longitudinal samples were obtained at lowland (day −23, i.e., 23 d before exposure to high altitude), followed by two time points at high altitude (days 14 and 20–Fig. 1*A*). Samples were also collected after returning to lowland (Grenoble and Lyon, France, approximately 250 m—5 lowland samples in total: day 2, 9, 16, 22, and 44 after return to low altitude). Analytical workflows are detailed in *SI Appendix*, *Supplementary Methods*.

## Results

Multivariate analyses indicated changes in all “omes” (Fig. 1 *A*–*F* and *SI Appendix*, *Supplementary Data*), with most notable altitude-associated elevations in BPG—the most significant result in the linear discriminant analysis in Fig. 1 *C* and *D*) and glycolytic metabolites. These results are consistent with the well-established role of BPG in hemoglobin allostery to promote oxygen off-loading (Fig. 1*G*). Changes in glycolysis were accompanied by a plethora of concomitant metabolic and lipidomics alterations, including activation of the Lands’ cycle, alterations in the levels and degree of unsaturation of free fatty acids and rewiring of arginine and polyamine metabolism (*SI Appendix*, *Supplementary Data*). Interestingly, linear discriminant analysis highlighted changes in the levels of the blood group RhC(E) polypeptide (RHCE—Fig. 1 *F* and *G*) and related subunits (RHD and RHAG). All six volunteers were Rh positive. The erythrocyte Rhesus factor complex is a heterotrimer of RhAG, RhD, and RhCE subunits in which RhD and RhCE might play roles in anchoring the ammonium-conducting RhAG subunit to the cytoskeleton (9). Ammonium (NH_4_^+^) is in equilibrium with ammonia (NH_3_) in aqueous solution. Given its high pKa (9.25), at typical cytosolic pH, the cationic form is the dominant species (~99%). However, while ammonia can pass through lipid bilayers along its concentration gradient, ammonium is orders of magnitude less permeant (10). Of note, both BPGM and phosphofructokinase activities are pH dependent, favoring glycolysis and the Rapoport–Luebering shunt at alkaline pH (11).

While the role of Rh antigens in transfusion-dependent blood group incompatible immune reactions and in hemolytic disease of the fetus and newborn has long been recognized, it is a relatively recent discovery that human Rh proteins share structural and functional homology with ammonium transporters in other species (e.g., Mep and AMT) (10). This is relevant in light of the genetic heterogeneity of Rh antigens in humans (12), a well-established concept in the field of transfusion medicine. Indeed, the CE/D subunits of the complex present the epitopes of the Rh antigens D or E/e and C/c on their external loops. The RhAG subunits are essential for expression of the whole complex, whereas the combination with RhCE and RhD varies. Two different genetic defects can lead to the rare Rh^null^ phenomenon: i) Mutations to RhAG prevent the translocation of the Rh complex to the cell surface, or ii) deletions of RhD are associated with simultaneous mutations to RhCE. While the Rh^null^ phenotype is rare, with a frequency of approximately 1 in 6 million individuals, approximately 15% of Caucasians are Rh D-negative, as compared to 1% individuals of Asian descent.

To delve further into the genetic underpinnings of BPG levels in RBCs, we measured BPG levels in 13,091 packed RBC units from blood donor volunteers enrolled in the Recipient Epidemiology and Donor evaluation Study (REDS) RBC Omics (https://biolincc.nhlbi.nih.gov/studies/reds_iii/). BPG levels were then used as a quantitative trait locus (QTL) to perform a metabolite QTL analysis (mQTL) by linking metabolite measurements to 879,000 single nucleotide polymorphisms (SNPs) that were monitored in this cohort. RBC BPG levels declined with donor age, were significantly higher in male donors, and were influenced by donor ethnicity, suggestive of a genetic effect (Fig. 1 *H*–*J*), whereas limited effects were observed as a function of BMI. Genome-wide association studies identified a significant association between BPG levels and SNPs on chromosome 1, in the region coding for RHCE (top SNP rs636889; *P* = 2.0 e-12–Fig. 1 *K* and *L*).

## Discussion

Our data suggest that genetic or environmental factors (e.g., altitude) modulate the expression of Rh group components, which in turn is associated with BPG levels in human RBCs. As a plausible mechanism, we hypothesize that expression of Rh blood group components may contribute to ammonium homeostasis and ultimately impact intracellular pH by promoting alkalinization, which would, in turn, boost BPGM activity and contribute to BPG generation (Fig. 1*M*). After identification of the mechanosensitive ion channel Piezo1 as a blood group–defining protein (13), we provide further evidence that blood group–defining proteins have functional impacts on RBC properties. Our results may have relevant translational implications for physiological and pathological states that rely on tight regulation of RBC oxygen kinetics by BPG levels beyond exposure to high altitude; for example, from exercise to cold exposure and from hemorrhagic or ischemic hypoxia to cardiorenal dysfunction.

## Supplementary Material

Appendix 01 (PDF)

Dataset S01 (XLSX)

## Data Availability

Metabolomics data have been deposited in Metabolomics Workbench (Embargoed until publication). All other data are included in the manuscript and/or supporting information.
