# Supplementary material for: Genetic polymorphisms and expression of Rhesus blood group RHCE are associated with 2,3-bisphosphoglycerate in humans at high altitude
Source: Proc Natl Acad Sci U S A. 2023 Dec 26;121(1):e2315930120. doi: 10.1073/pnas.2315930120 (PMC10769835; doi:10.1073/pnas.2315930120)
Supplement: Supplementary file 1 — Appendix 01 (PDF) [file pnas.2315930120.sapp.pdf]

Genetic polymorphisms and expression of Rhesus blood group RHCE are associated with 2,3-bisphosphoglycerate in humans at high altitude

Angelo D'Alessandro,[1,2*] Eric Earley,[3] Travis Nemkov,[1,2] Daniel Stephenson,[1] Monika Dzieciatkowska,[1] Kirk Hansen,[1] Giampaolo Minetti,[4] Benoit Champigneulle[5], Emeric Stauffer[6], Aurélien Pichon[7], Michael Furian[8], Samuel Verges, [5] Steven Kleinman, [9] Philip Norris,[10] Michael P. Busch, [10] Grier P. Page,[3] Lars Kaestner[11,12,*]

1) Department of Biochemistry and Molecular Genetics, University of Colorado Denver – Anschutz Medical Campus, Aurora, CO, USA;
2) Omix Technologies Inc, Aurora, CO, USA;
3) RTI International, Atlanta, GA, USA;
4) Department of Biology and Biotechnology, University of Pavia, Italy;
5) Hypoxia Physiopathology laboratory (HP2), INSERM U1042, Grenoble Alpes University, Grenoble, France;
6) Laboratoire Interuniversitaire de Biologie de la Motricité (LIBM) EA7424, Université Claude Bernard Lyon 1, Université de Lyon, Explorations Fonctionnelles Respiratoires, Médecine du sport et de l'Activité Physique, Hospices Civils de Lyon, Hôpital Croix Rousse, Lyon, France
7) Université de Poitiers, Laboratoire MOVE, Poitiers, France
8) Pulmonology Department, University of Zurich, Zürich, Switzerland
9) University of British Columbia, Victoria, British Columbia, Canada;
10) Vitalant Research Institute, San Francisco CA, USA;
11) Theoretical Medicine and Biosciences, Saarland University, Homburg, Germany;
12) Dynamics of Fluids, Experimental Physics, Saarland University, Saarbrücken, Germany.

***Corresponding author:**
Angelo D'Alessandro, PhD
Department of Biochemistry and Molecular Genetics
University of Colorado Anschutz Medical Campus
12801 East 17th Ave., Aurora, CO 80045
Phone # 303-724-0096
E-mail: angelo.dalessandro@ucdenver.edu

Lars Kaestner, PhD
Experimental Physics and Theoretical Medicine and Biosciences
Saarland University
Saarbrücken, Germany
E-mail: lars_kaestner@me.com

TABLE OF CONTENTS

SUPPLEMENTARY MATERIALS AND METHODS EXTENDED 2
SUPPLEMENTARY METHODS - REFERENCES 4
SUPPLEMENTARY DATA .... XLS

Supplementary Materials and Methods - Extended

Expedition 5300: Proteomics analyses Proteomics analyses were performed as described (1). A volume of 10 µL of RBCs were lysed in 90 µL of distilled water; 5 µL of lysed RBCs were mixed with 45 µL of 5% SDS and then vortexed. Samples were reduced with 10 mM DTT at 55 °C for 30 min, cooled to room temperature, and then alkylated with 25 mM iodoacetamide in the dark for 30 min. Next, a final concentration of 1.2% phosphoric acid and then six volumes of binding buffer (90% methanol; 100 mM triethylammonium bicarbonate, TEAB; pH 7.1) were added to each sample. After gentle mixing, the protein solution was loaded to a S-Trap 96-well plate, spun at 1500 x g for 2 min, and the flow-through collected and reloaded onto the 96-well plate. This step was repeated three times, and then the 96-well plate was washed with 200 µL of binding buffer 3 times. Finally, 1 µg of sequencing-grade trypsin (Promega) and 125 µL of digestion buffer (50 mM TEAB) were added onto the filter and digested carried out at 37 °C for 6 h. To elute peptides, three stepwise buffers were applied, with 100 µL of each with one more repeat, including 50 mM TEAB, 0.2% formic acid (FA), and 50% acetonitrile and 0.2% FA. The peptide solutions were pooled, lyophilized, and resuspended in 500 µL of 0.1 % FA.

Each sample was loaded onto individual Evotips for desalting and then washed with 200 µL 0.1% FA followed by the addition of 100 µL storage solvent (0.1% FA) to keep the Evotips wet until analysis. The Evosep One system (Evosep, Odense, Denmark) was used to separate peptides on a Pepsep column, (150 um inter diameter, 15 cm) packed with ReproSil C18 1.9 um, 120A resin. The system was coupled to a timsTOF Pro mass spectrometer (Bruker Daltonics, Bremen, Germany) via a nano-electrospray ion source (Captive Spray, Bruker Daltonics). The mass spectrometer was operated in PASEF mode. The ramp time was set to 100 ms and 10 PASEF MS/MS scans per topN acquisition cycle were acquired. MS and MS/MS spectra were recorded from *m/z* 100 to 1700. The ion mobility was scanned from 0.7 to 1.50 Vs/cm2. Precursors for data-dependent acquisition were isolated within ± 1 Th and fragmented with an ion mobility-dependent collision energy, which was linearly increased from 20 to 59 eV in positive mode. Low-abundance precursor ions with an intensity above a threshold of 500 counts but below a target value of 20000 counts were repeatedly scheduled and otherwise dynamically excluded for 0.4 min.

Database Searching and Protein Identification MS/MS spectra were extracted from raw data files and converted into .mgf files using MS Convert (ProteoWizard, v. 3.0). Peptide spectral matching was performed with Mascot (v. 2.5) against the Uniprot human database. Mass tolerances were +/- 15 ppm for parent ions, and +/- 0.4 Da for fragment ions. Trypsin specificity was used, allowing for 1 missed cleavage. Met oxidation, Cys dioxidation, Cys thiol conversion to dehydroalanine, protein N-terminal acetylation, isopeptide bond formation with loss of ammonia (K), and peptide N-terminal pyroglutamic acid formation were set as variable modifications with Cys carbamidomethylation set as a fixed modification.

Scaffold (v 4.8, Proteome Software, Portland, OR, USA) was used to validate MS/MS based peptide and protein identifications. Peptide identifications were accepted if they could be established at greater than 95.0% probability as specified by the Peptide Prophet algorithm. Protein identifications were accepted if they could be established at greater than 99.0% probability and contained at least two identified unique peptides.

Oxidized cysteine content was determined by summing the spectral counts for peptides containing Cys sulfinic acid (Cys-SO_2H, i.e. deoxidation) and Cys conversion to dehydroalanine, a stable end-

product of oxidized cysteine intermediates. The irreversibly oxidized Cys content was normalized to total peptide spectral counts for the corresponding protein. Asparagine deamidation and glutamate/aspartate methylation were determined using database searches with these variable modifications. Results were normalized to total peptide spectral counts for the corresponding protein.

Expedition 5300: Lipidomics analyses Total lipids were extracted as previously described (2): 10 µL of RBCs were mixed with 90 µL of cold methanol. Samples were then briefly vortexed and incubated at -20 °C for 30 minutes. Following incubation, samples were centrifuged at 12,700 RPM for 10 minutes at 4 °C and 80 µL of supernatant was transferred to a new tube for analysis. Lipid extracts were analyzed (10 uL per injection) on a Thermo Vanquish UHPLC/Q Exactive MS system using a 5 min lipidomics gradient and a Kinetex C18 column (30 x 2.1 mm, 1.7 µm, Phenomenex) held at 50 °C. Mobile phase A: 25:75 MeCN:water with 5 mM ammonium acetate; Mobile phase B: 90:10 isopropanol:MeCN with 5 mM ammonium acetate. The gradient and flow rate were as follows: 0.3 mL/min of 10% B at 0 min, 0.3 mL/min of 95% B at 3 min, 0.3 mL/min of 95% B at 4.2 min, 0.45 mL/min 10% B at 4.3 min, 0.4 mL/min of 10% B at 4.9 min, and 0.3 mL/min of 10% B at 5 min. Samples were run in positive and negative ion modes (both ESI, separate runs) at 125 to 1500 m/z and 70,000 resolution, 4 kV spray voltage, 45 sheath gas, 25 auxiliary gas. The MS was run in data-dependent acquisition mode ($ddMS^2$) with top10 fragmentation. Raw MS data files were searched using LipidSearch v 5.0 (Thermo).

Donor recruitment in the REDS RBC Omics study A total of 13,758 donors were enrolled in the Recipient Epidemiology and Donor evaluation Study (REDS) RBC Omics at four different blood centers across the United States (https://biolincc.nhlbi.nih.gov/studies/reds_iii/). Of these, 97% (13,403) provided informed consent and 13,091 were available for metabolomics analyses in this study – henceforth referred to as "index donors". Metabolomics analyses were performed on leukocyte-filtered packed RBCs derived from whole blood donations from this cohort.(3)

High-throughput metabolomics (for both Expedition 5300 and REDS RBC Omics) Metabolomics extractions and analyses in 96 well-plate format were performed as extensively described in previous studies (4, 5). RBC samples were transferred on ice on 96 well plate and frozen at -80 ºC at Vitalant San Francisco prior to shipment in dry ice to the University of Colorado Anschutz Medical Campus. Plates were thawed on ice then a 10 µL aliquot was transferred with a multi-channel pipettor to 96-well extraction plates. A volume of 90 µL of ice cold 5:3:2 MeOH:MeCN:water (*v/v/v*) was added to each well, with an electronically-assisted cycle of sample mixing repeated three times. Positive pressure was then applied via a 96-well plate manifold via N_2, which facilitated sample extract release into the sample plate. Each plate was covered with a sealed mat and transferred to an ultra-high-pressure liquid chromatography (UHPLC-MS — Vanquish) equipped with a plate charger. A blank containing a mix of standards detailed before (6) and a quality control sample (the same across all plates) were injected 5 times each per plate and used to monitor instrument performance through the analysis. Metabolites were resolved on a Phenomenex Kinetex C18 column (2.1 x 150 mm, 1.7 µm) at 45 °C using a 1-minute ballistic gradient method in positive and negative ion modes (separate runs) over the scan range 65-975 m/z exactly as previously described (4). The UHPLC was coupled online to a Q Exactive mass spectrometer (Thermo Fisher). The Q Exactive MS was operated in negative ion mode, scanning

in Full MS mode (2 µscans) from 90 to 900 m/z at 70,000 resolution, with 4 kV spray voltage, 45 sheath gas, 15 auxiliary gas. Following data acquisition, .raw files were converted to .mzXML using RawConverter then metabolites assigned and peaks integrated using ElMaven (Elucidata) in conjunction with an in-house standard library (7).

mQTL analysis The workflow for the 2,3-bisphosphoglycerate (BPG) mQTL analysis is consistent with previously described methods from our pilot mQTL study on 250 recalled donors (8). Details of the genotyping and imputation of the RBC Omics study participants have been previously described by Page, et al. (9) Briefly, genotyping was performed using a Transfusion Medicine microarray (10) and the data are available in dbGAP accession number phs001955.v1.p1. Imputation was performed using 811,782 SNPs that passed quality control. After phasing using Shape-IT (11), imputation was performed using Impute2 (12) with the 1000 Genomes Project phase 3 (12) all-ancestry reference haplotypes. We used the R package SNPRelate (13) to calculate principal components (PCs) of ancestry. We performed association analyses for L-carnitine and acyl-carnitines using an additive SNP model in the R package ProbABEL (14) and 13,091 study participants who had both metabolomics data and imputation data on serial samples from stored RBC components that passed respective quality control procedures. We adjusted for sex, age (continuous), frequency of blood donation in the last two years (continuous), blood donor center, and ten ancestry PCs. Statistical significance was determined using a p-value threshold of $5x10^{-8}$, adjusted for multiple observations. For example, through this analysis we identified SNPs following a classic "train" structure in the Manhattan plot representation of the findings. These SNPs mapped on a region of chromosome 1 coding for EDEM2. However, these results did not reach FDR-adjusted significance and are thus not discussed in the brief report. Other variants are noted in the locus zoom in regions flanking the top SNPs (e.g., MACO1 – >10 fold less significant than the top SNP rs636889). MACO1 codes for the macoilin 1 protein, which plays a role in the regulation of neuronal activity, thus making it biologically less likely to be associated with RBC metabolic responses to altitude (i.e., altered rates of BPG synthesis). We only considered variants with a minimum minor allele frequency of 1% and a minimum imputation quality score of 0.80. The OASIS: Omics Analysis, Search & Information a TOPMED funded resources (15), was used to annotate the top SNPS. OASIS annotation includes information on position, chromosome, alellele frequencies, closest gene, type of variant, position relative to closest gene model, if predicted to functionally consequential, tissues specific gene expression, and other information.

Data analysis Statistical analyses – including hierarchical clustering analysis (HCA), linear discriminant analysis (LDA), hierarchical clustering analyses (HCA), time series ANOVA and correlation analyses were performed using both MetaboAnalyst 5.0 (16) and in-house developed code in R (4.2.3 2023-03-15).

Supplementary references

1. T. Thomas *et al.*, Evidence of Structural Protein Damage and Membrane Lipid Remodeling in Red Blood Cells from COVID-19 Patients. *J Proteome Res* **19**, 4455-4469 (2020).

2. J. A. Reisz, C. Zheng, A. D'Alessandro, T. Nemkov, Untargeted and Semi-targeted Lipid Analysis of Biological Samples Using Mass Spectrometry-Based Metabolomics. *Methods Mol Biol* **1978**, 121-135 (2019).
3. A. D'Alessandro *et al.*, Heterogeneity of blood processing and storage additives in different centers impacts stored red blood cell metabolism as much as storage time: lessons from REDS-III-Omics. *Transfusion* **59**, 89-100 (2019).
4. T. Nemkov, T. Yoshida, M. Nikulina, A. D'Alessandro, High-Throughput Metabolomics Platform for the Rapid Data-Driven Development of Novel Additive Solutions for Blood Storage. *Front Physiol* **13**, 833242 (2022).
5. T. Nemkov, J. A. Reisz, S. Gehrke, K. C. Hansen, A. D'Alessandro, High-Throughput Metabolomics: Isocratic and Gradient Mass Spectrometry-Based Methods. *Methods Mol Biol* **1978**, 13-26 (2019).
6. D. Stefanoni *et al.*, Red blood cell metabolism in Rhesus macaques and humans: comparative biology of blood storage. *Haematologica* **105**, 2174-2186 (2020).
7. T. Nemkov, K. C. Hansen, A. D'Alessandro, A three-minute method for high-throughput quantitative metabolomics and quantitative tracing experiments of central carbon and nitrogen pathways. *Rapid communications in mass spectrometry : RCM* **31**, 663-673 (2017).
8. A. Moore *et al.*, Genome-wide metabolite quantitative trait loci analysis (mQTL) in red blood cells from volunteer blood donors. *J Biol Chem* **298**, 102706 (2022).
9. G. P. Page *et al.*, Multiple-ancestry genome-wide association study identifies 27 loci associated with measures of hemolysis following blood storage. *J Clin Invest* **131** (2021).
10. Y. Guo *et al.*, Development and evaluation of a transfusion medicine genome wide genotyping array. *Transfusion* **59**, 101-111 (2019).
11. O. Delaneau, C. Coulonges, J.-F. Zagury, Shape-IT: new rapid and accurate algorithm for haplotype inference. *BMC Bioinformatics* **9**, 540 (2008).
12. B. Howie, J. Marchini, M. Stephens, Genotype Imputation with Thousands of Genomes. *G3 Genes|Genomes|Genetics* **1**, 457-470 (2011).
13. X. Zheng *et al.*, A high-performance computing toolset for relatedness and principal component analysis of SNP data. *Bioinformatics* **28**, 3326-3328 (2012).
14. Y. S. Aulchenko, M. V. Struchalin, C. M. van Duijn, ProbABEL package for genome-wide association analysis of imputed data. *BMC Bioinformatics* **11**, 134 (2010).
15. J. A. Perry, B. J. Gaynor, B. D. Mitchell, J. R. O’Connell, An Omics Analysis Search and Information System (OASIS) for Enabling Biological Discovery in the Old Order Amish. *bioRxiv* 10.1101/2021.05.02.442370, 2021.2005.2002.442370 (2021).
16. Z. Pang *et al.*, MetaboAnalyst 5.0: narrowing the gap between raw spectra and functional insights. *Nucleic Acids Research* **49**, W388-W396 (2021).
